# Antiviral activities of Indonesian medicinal plants in the East Java region against hepatitis C virus

**DOI:** 10.1186/1743-422X-10-259

**Published:** 2013-08-13

**Authors:** Tutik Sri Wahyuni, Lydia Tumewu, Adita Ayu Permanasari, Evhy Apriani, Myrna Adianti, Abdul Rahman, Aty Widyawaruyanti, Maria Inge Lusida, Achmad Fuad, Soetjipto, Nasronudin, Hiroyuki Fuchino, Nobuo Kawahara, Ikuo Shoji, Lin Deng, Chie Aoki, Hak Hotta

**Affiliations:** 1 Department of Pharmacognocy and Phytochemistry, Faculty of Pharmacy, Airlangga University, Surabaya, Indonesia; 2 Institute of Tropical Disease, Airlangga University, Surabaya, Indonesia; 3 Division of Microbiology, Kobe University Graduate School of Medicine, Kobe, Japan; 4 Research Center for Medicinal Plant Resources, National Institute of Biomedical Innovation, Tsukuba, Ibaraki, Japan; 5 JST/JICA SATREPS, Kobe University Graduate School of Medicine, Kobe, Japan

**Keywords:** Hepatitis C virus, HCV, Antiviral activity, Medicinal plants, Indonesia, Entry inhibition

## Abstract

**Background:**

Hepatitis C virus (HCV) is a major cause of liver disease and a potential cause of substantial morbidity and mortality worldwide. The overall prevalence of HCV infection is 2%, representing 120 million people worldwide. Current standard treatment using pegylated interferon and ribavirin is effective in only 50% of the patients infected with HCV genotype 1, and is associated with significant side effects. Therefore, it is still of importance to develop new drugs for treatment of HCV. Antiviral substances obtained from natural products, including medicinal plants, are potentially good targets to study. In this study, we evaluated Indonesian medicinal plants for their anti-HCV activities.

**Methods:**

Ethanol extracts of 21 samples derived from 17 species of medicinal plants explored in the East Java region were tested. Anti-HCV activities were determined by a cell culture method using Huh7.5 cells and HCV strains of 9 different genotypes (1a to 7a, 1b and 2b).

**Results:**

Four of the 21 samples tested showed antiviral activities against HCV: *Toona sureni* leaves (TSL) with 50% inhibitory concentrations (IC_50_) of 13.9 and 2.0 μg/ml against the HCV J6/JFH1-P47 and -P1 strains, respectively, *Melicope latifolia* leaves (MLL) with IC_50_ of 3.5 and 2.1 μg/ml, respectively, *Melanolepis multiglandulosa* stem (MMS) with IC_50_ of 17.1 and 6.2 μg/ml, respectively, and *Ficus fistulosa* leaves (FFL) with IC_50_ of 15.0 and 5.7 μg/ml, respectively. Time-of-addition experiments revealed that TSL and MLL inhibited both at the entry and post-entry steps while MMS and FFL principally at the entry step. TSL and MLL inhibited all of 11 HCV strains of all the genotypes tested to the same extent. On the other hand, FFL showed significantly weaker inhibitory activities against the HCV genotype 1a strain, and MMS against the HCV strains of genotypes 2b and 7a to a lesser extent, compared to the other HCV genotypes.

**Conclusions:**

Ethanol extracts of TSL, MLL, MMS and FFL showed antiviral activities against all the HCV genotypes tested with the exception that some genotype(s) showed significant resistance to FFL and to MMS to a lesser extent. These plant extracts may be good candidates for the development of anti-HCV drugs.

## Background

Hepatitis C virus (HCV) is an enveloped virus that belongs to the *Hepacivirus* genus within the *Flaviviridae* family. The viral genome is a single-stranded, positive-sense RNA of 9.6 kb with highly structured 5’- and 3’-untranslated regions [1]. It encodes a polyprotein precursor consisting of about 3,000 amino acid residues, which is cleaved by the host and viral proteases to generate 10 mature proteins, such as core, E1, E2, a putative ion channel p7, and nonstructural proteins NS2, NS3, NS4A, NS4B, NS5A and NS5B [1,2]. Core, E1 and E2 together with the viral genome form the infectious virus particle while the other nonstructural proteins are essential for viral RNA replication. The HCV genome exhibits a considerable degree of sequence heterogeneity, based on which HCV is currently classified into 7 genotypes (1 to 7) and more than 70 subtypes (1a, 1b, 2a, 2b, etc.) [3].

HCV is a major cause of chronic liver disease, such as hepatitis, liver cirrhosis and hepatocellular carcinoma, and is a potential cause of substantial morbidity and mortality [4,5]. The most recent estimate of the prevalence of HCV infection reported by the World Health Organization is 2%, representing 120 million people worldwide. A current standard treatment using pegylated interferon and ribavirin is effective in only ca. 50% of the patients infected with HCV genotype 1, and is associated with significant side effects and viral resistance [3]. Although a number of novel antivirals against HCV for clinical use are being tested, it is still of importance to develop complementary and/or alternative drugs for treatment of HCV infection from clinical and economical points of view. In this regard, antiviral substances obtained from natural products, including medicinal plants, are potentially good targets to study [6].

It is well known that certain medicinal plants possess antiviral activities. A wide variety of active phytochemicals, such as flavonoids, terpenoids, lignins, sulphides, polyphenolics, coumarins, saponins, furyl compounds, alkaloids, polylines, thiophenes, proteins and peptides, have been identified to inhibit various viruses [7]. Herbal extracts of *Boswellia carterii, Embelia schimperi, Piper cubeba, Quercus infectoria, Tranchyspermum ammi* and *Syzygium aromaticum* were shown to inhibit HCV protease activities *in vitro*[8]. A methanol extract of *Swietenia macrophilla* stem and a purified compound, 3-hydroxy caruilignan, inhibited HCV RNA replication in Huh7 cells harboring an HCV subgenomic RNA replicon [9]. Also, inhibition of HCV replication by herbal extracts was reported on leaves and roots of *Phyllantus amarus* (Euphorbiaceae) [10]. Moreover, a number of bioflavonoid compounds, such as catechin, narigenin and quercetin, significantly inhibited HCV replication [11], with quercetin inhibiting the HCV NS3 serine protease activity [12]. Further studies to identify antiviral activities of medicinal plants offer a great opportunity to find effective new drug candidates. Indonesia is said to possess the second largest biodiversity in the world, with around 40,000 endemic plant species including 6,000 medicinal plants [13]. In this study, ethanol extracts of certain Indonesian medicinal plants explored from the East Java region were evaluated for their anti-HCV activities.

## Results

### Anti-HCV activities of ethanol extracts of Indonesian medicinal plants

**Figure 1:**
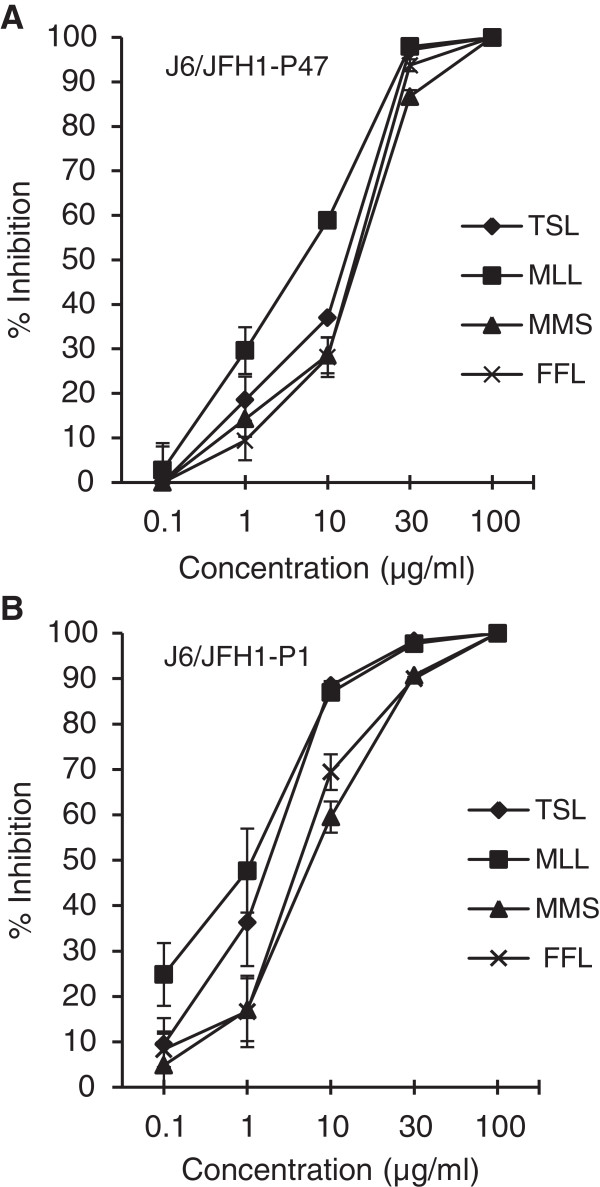
**Dose-dependent inhibition of HCV infection by ethanol extracts of TSL, MLL, MMS and FFL.** The HCV J6/JFH1-P47 **(A)** and -P1 strains **(B)** were mixed with serial dilutions of the plant extracts and inoculated to Huh 7.5 cells at an MOI of 0.5 and 0.05, respectively. After virus adsorption, the cells were cultured with the same concentrations of plant extracts for 46 hours. The culture supernatants were harvested and titrated for the virus infectivity. Percent inhibitions of HCV infectivity by the plant extracts at the concentrations of 0.1 to 100 μg/ml are shown. Data represent means ± SEM of data from two independent experiments.

A total of 21 samples from 17 species of medicinal plants explored in the East Java region, Indonesia, were used in this study. The botanical names, the families and the parts of the plants were verified by botanists. Ethanol extracts of the plants were examined for antiviral activities against the J6/JFH1-P47 (passage 47) strain of HCV genotype 2a [14] in a cell culture system using Huh7.5 cells at a multiplicity of infection (MOI) of 0.5. The 50% inhibitory concentrations (IC_50_), the 50% cytotoxic concentrations (CC_50_) and selectivity indexes (SI: CC_50_/IC_50_) of the plant extracts are shown in Table 1. The results obtained revealed that 4 of the 21 extracts possessed potential anti-HCV activities against HCV J6/JFH1-P47 with IC_50_ being <20 μg/ml and CC_50_ being >100 μg/ml. The positive samples were: *Toona sureni* leaves (TSL; IC_50_ = 13.9 μg/ml)*, Melicope latifolia* leaves (MLL; IC_50_ = 3.5 μg/ml)*, Melanolepis multiglandulosa* stem (MMS; IC_50_ = 17.1 μg/ml) and *Ficus fistulosa* leaves (FFL; IC_50_ = 15.0 μg/ml). Dose-dependent anti-HCV activities of TSL, MLL, MMS and FFL extracts against the HCV J6/JFH1-P47 [14] were shown in Figure 1A.

**Table 1:** **Antiviral activity (IC**_**50**_**) against HCV J6/JFH1-P47, cytotoxicity (CC**_**50**_**) and selectivity index (SI) of Indonesian medicinal plants tested in this study**

**No.**	**Botanical name**	**Parts**	**Family**	**IC**_**50**_^**a **^**(μg/ml)**	**CC**_**50 **_**(μg/ml)**	**SI**
1.	*Eupatorium inulifolium*	Stems	Asteraceae	> 500	>500	na^b^
2.	*Calliandra polytirsa*	Leaves	Fabaceae	31.9 ± 7.1	>100	>3.1
3.	*Strophacantus membranifolius*	Herbs	Acantaceae	>100	>500	na
4.	*Cestrum calysinum*	Leaves	Solanaceae	52.1 ± 5.7	>500	>9.6
5.	*Cestrum calysinum*	Stems	Solanaceae	>500	>500	na
6.	*Eucalyptus globulus*	Stems	Myrtaceae	43.0 ± 39.5	>100	>2.3
7.	***Toona sureni***^***c***^	Leaves	Meliaceae	**13.9** ± **1.6**	**> 500**	**>35.9**
8.	***Melicope latifolia***^***c***^	Leaves	Rutaceae	**3.5** ± **1.4**	**>100**	**>28.6**
9.	*Melicope latifolia*	Stems	Rutaceae	42.6 ± 37.6	>100	>2.4
10.	*Piper sulcatum*	Stems	Piperaceae	38.0 ± 4.2	>100	>2.6
11.	*Fagraea blumei*	Stems	Fagaceae	>100	>500	na
12.	*Fraxinus griffithii*	Stems	Meliaceae	>500	>500	na
13.	*Maesa latifolia*	Leaves	Myrsinaceae	32.7 ± 6.6	>100	>3.1
14.	*Maesa latifolia*	Stems	Myrsinaceae	32.2 ± 10.2	>100	>3.1
15.	***Melanolepis multiglandulosa***^***c***^	Stems	Euphorbiaceae	**17.1** ± **1.6**	**>100**	**>5.8**
16.	*Acacia decurens*	Leaves	Fabaceae	44.9 ± 7.1	>500	>11.1
17.	*Randia maculata*	Stems	Rubiaceae	38.7 ± 5.7	>500	>12.9
18.	*Gompostemma polythirsa*	Flowers	Acanthaceae	92.8 ± 19.8	>500	>5.4
19.	*Acmena acuminatissima*	Leaves	Myrtaceae	>100	>100	na
20.	*Acmena acuminatissima*	Stems	Myrtaceae	>100	>500	na
21.	***Ficus fistulosa***^***c***^	Leaves	Moraceae	**15.0** ± **7.1**	**>100**	**>7.6**

^a^Data represent means ± SEM of data from two independent experiments using HCV J6/JFH1-P47.

^b^Not applicable.

^c^The plant extracts with IC_50_ of <20 μg/ml and CC_50_ of >100 μg/ml are written in boldface letters.

### Mode of action of ethanol extracts of TSL, MLL, MMS and FFL

To determine whether the anti-HCV effects of TSL, MLL, MMS and FFL extracts are exerted at the entry or the post-entry step, time-of-addition experiments were performed, in which three sets of experiments were done in parallel: (i) HCV was mixed with a plant extract (30 μg/ml) and the mixture was inoculated to the cells. After virus adsorption for 2 hours, the residual virus and the plant extract were removed, and cells were refed with fresh medium without the plant extract for 46 hours. This experiment examines the antiviral effect at the entry step. (ii) HCV was inoculated to the cells in the absence of the plant extract. After virus adsorption for 2 hours, the residual virus was removed and cells were refed with fresh medium containing the plant extract (30 μg/ml) for 46 hours. This experiment examines the antiviral effect at the post-entry step. (iii) As a positive control, HCV mixed with the plant extract was inoculated to the cells. After virus adsorption for 2 hours, the residual virus and the plant extract were removed, and cells were refed with fresh medium containing the plant extract for 46 hours. As shown in Table 2, ethanol extracts of TSL and MLL showed anti-HCV activities at both the entry and post-entry steps. On the other hand, MMS and FFL exhibited anti-HCV activities principally at the entry step.

**Table 2:** **Mode of action of ethanol extracts of *T. sureni *leaves (TSL), *M. latifolia *leaves (MLL), *M. multiglandulosa *stem (MMS) and *F. fistulosa *leaves (FFL)**

**Plant extract**	**% Inhibition**^**a**^			**Mode of action**
	**During + Post**	**During**	**Post**	
	**inoculation**	**inoculation**	**inoculation**	
*T. sureni* leaves (TSL)	97.2 ± 1.3^b^	92.2 ± 2.2	60.9 ± 2.2	Entry inhibition
				Post-entry inhibition
*M. latifolia* leaves (MLL)	98 ± 0.3	90.8 ± 0.2	60.6 ± 4.9	Entry inhibition
				Post-entry inhibition
*M. multiglandulosa* stem (MMS)	86.6 ± 1.4	73 ± 0.9	33.5 ± 1.4	Entry inhibition
*F.fistulosa* leaves (FFL)	93.8 ± 1.3	86.7 ± 3.1	20.5 ± 2.6	Entry inhibition

^a^% Inhibition at the concentration of 30 μg/ml.

^b^Data represent means ± SEM of data from two independent experiments using HCV J6/JFH1-P47.

**Figure 2:**
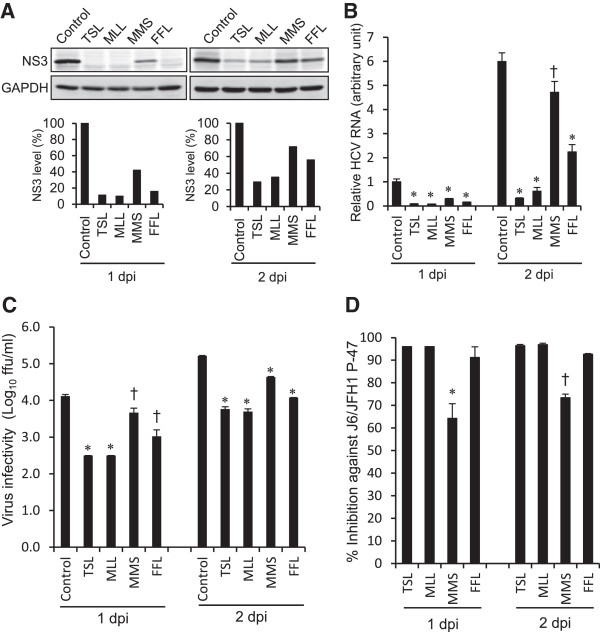
**Inhibition of HCV protein expression, HCV RNA replication and infectious virus production by ethanol extracts of TSL, MLL, MMS and FFL. (A)** Huh 7.5 cells infected with HCV J6/JFH1-P47 and treated with the extracts (30 μg/ml) of TSL, MLL, MMS and FFL (see Figure 1A) and the untreated control were subjected to Western blot analysis using monoclonal antibody against the HCV NS3 protein at 1 and 2 days post-infection (dpi). GAPDH served as an internal control to verify equal amounts of sample loading. Signal intensities of NS3 were normalized to the corresponding GAPDH signal. **(B)** Amounts of HCV RNA in the cells described in **(A)** were measured by real-time quantitative RT-PCR analysis. The HCV RNA amounts were normalized to GAPDH mRNA expression levels. Data represent means ± SEM of data from two independent experiments, and the value for the untreated control at 1 dpi was arbitrarily expressed as 1.0. *, *P* < 0.000001; ǂ, *P* < 0.001, compared with the control. **(C)** Virus infectivity in the culture supernatants of the cells described in (A) was measured. Data represent means ± SEM of data from two independent experiments. ǂ, *P* <0.05; *, *P* < 0.005, compared with the control. **(D)** Inhibition of HCV infectivity by the extracts (30 μg/ml) of TSL, MLL, MMS and FFL are shown. *, *P* < 0.05; ǂ, *P* < 0.01, compared with TSL.

To further confirm anti-HCV activities of the extracts of TSL, MLL, MMS and FFL, we investigated whether those extracts (30 μg/ml) affect HCV protein expression level, HCV RNA replication and infectious virus production in HCV J6/JFH1-P47-infected cells. The results showed that treatment with TSL and MLL markedly decreased the amounts of the HCV NS3 protein while that with MMS and FFL to lesser extents (Figure 2A). To quantitate the effect of TSL, MLL, MMS and FFL more accurately, we measured HCV RNA levels by real-time quantitative RT-PCR. Again, TSL and MLL markedly suppressed HCV RNA levels while MMS and FFL to lesser extents (Figure 2B). Moreover, TSL and MLL markedly inhibited the infectious virus production by >1 log_10_, FFL by 1 log_10_ while MMS to a lesser extent of <1 log_10_ (Figure 2C and 2D).

### Antiviral activities of ethanol extracts of TSL, MLL, MMS and FFL against HCV genotypes 1 to 7

Antiviral activities of the extracts of TSL, MLL, MMS and FFL were further examined for other HCV strains of various genotypes. First, we examined the HCV J6/JFH1-P1 (passage 1) strain [15] and found that TSL, MLL, MMS and FFL inhibited HCV J6/JFH1-P1 infection with IC_50_ of 2.0, 2.1, 6.2 and 5.7 μg/ml, respectively. Dose-dependent anti-HCV activities of those extracts against the HCV J6/JFH1-P1 were shown in Figure 1B. We then compared anti-HCV activities of those plant extracts (30 μg/ml) using other HCV genotypes, including 1a to 7a, 1b and 2b [3] along with the JFH1 strain of genotype 2a [16]. The results showed that TSL and MLL exerted antiviral activities against all the HCV strains tested almost to the same extent (Table 3). On the other hand, MMS exhibited significantly weaker antiviral activities against the J8/JFH1 and QC69/JFH1 strains of genotypes 2b and 7a, respectively, compared to the other HCV strains. Notably, FFL at the concentration of 30 μg/ml did not exert detectable antiviral activities against the H77C/JFH1 strain of genotype 1a while exhibiting >90% inhibition at the same concentration against all the other HCV strains tested.

**Table 3:** **Antiviral activities of ethanol extracts of *T. sureni *leaves (TSL), *M. latifolia *leaves (MLL), *M. multiglandulosa *stem (MMS) and *F.fistulosa *leaves (FFL) against various HCV strains of different genotypes**

**HCV strain (genotype)**	**% Inhibition**^**a**^			
	***T. sureni *leaves**	***M. latifolia *leaves**	***M. multiglandulosa *stem**	***F. fistulosa *leaves**
	**(TSL)**	**(MLL)**	**(MMS)**	**(FFL)**
J6/JFH1 P47 (2a)	97.2 ± 1.3^b^	98.0 ± 0.3	86.7 ± 1.4	93.8 ± 1.3
J6/JFH1 (2a)	98.5 ± 2.1	99.5 ± 0.7	73.2 ± 2.1	96.1 ± 1.4
JFH1 (2a)	100 ± 0.0	100.0 ± 0.0	67.3 ± 8.2	92.3 ± 0.0
H77C/JFH1 (1a)	98.2 ± 2.5	100.0 ± 0.0	83.9 ± 2.5	**5.4 ± 2.5**^**c**^
J4/JFH1 (1b)	79.2 ± 11.8	91.7 ± 5.9	72.9 ± 8.8	93.8 ± 2.9
J8/JFH1 (2b)	100.0 ± 0.0	100.0 ± 0.0	**34.4 ± 13.3**^**c**^	93.8 ± 0.0
S52/JFH1 (3a)	97.8 ± 3.1	100.0 ± 0.0	95.6 ± 3.1	97.8 ± 0.0
ED43/JFH1 (4a)	94.2 ± 1.2	98.3 ± 0.0	59.5 ± 1.2	97.5 ± 1.2
SA13/JFH1 (5a)	100.0 ± 0.0	96.1 ± 1.1	89.8 ± 1.1	93.8 ± 2.2
HK6a/JFH1 (6a)	100.0 ± 0.0	91.2 ± 4.2	67.6 ± 20.8	91.2 ± 4.2
QC69/JFH1 (7a)	100.0 ± 0.0	87.0 ± 6.1	**39.1 ± 0.0**^**c**^	95.7 ± 6.1

^a^% Inhibition at the concentration of 30 μg/ml.

^b^Data represent means ± SEM of data from two independent experiments using HCV J6/JFH1-P47.

^c^% Inhibition of <40% are written in boldface letters.

## Discussion

A wide variety of traditional medicinal plants and herbs were reported to have antiviral activities against various viruses. In this study we analyzed anti-HCV activities of ethanol extracts of 21 medicinal plants that belong to 17 different species explored in the East Java region, Indonesia. In the initial screening, we used the HCV J6/JFH1-P47 strain as it is highly adapted to the Huh7.5 cell culture system [14] and, therefore, was easier to apply for the screening of many samples than the original P1 strain. Once we found possible candidates with anti-HCV activities, we used the original J6/JFH1-P1 strain to confirm the results.

Of the 21 samples, *T. sureni* leave (TSL), *M. latifolia* leave (MLL), *M. multiglandulosa* stem (MMS) and *F. fistulosa* leaves (FFL) were found to possess significant anti-HCV activities with IC_50_ of 13.9, 3.5, 17.1 and 15.0 μg/ml, respectively, against the J6/JFH1-P47 strain of HCV genotype 2a (Table 1 and Figure 1A), and 2.0, 2.1, 6.2 and 5.7 μg/ml, respectively, against the J6/JFH1-P1 strain (Figure 1B). We further examined anti-HCV activities of those plant extracts against other HCV genotypes, including 1a to 7a, 1b and 2b [3]. Although most of the HCV strains of different genotypes tested were inhibited by those plant extracts, there were some exceptions; the H77C/JFH1 strain (genotype 1a) showed significant resistance to FFL, and the J8/JFH1 (2b) and QC69/JFH1 strains (7a) to MMS to a lesser extent (Table 3). The difference in the amino acid sequences of the viral envelope proteins, especially E2, is likely to account for the different degree of the inhibition by a given extract among different HCV strains.

In this study we have not yet isolated a compound responsible for the anti-HCV activities; the study is still under way. It was reported that a methanol extract of *T. sureni*, a plant from Meliaceae family, showed antiviral activities against herpes simplex virus type 1 (HSV-1) with IC_50_ of 37 μg/ml [17]. By activity-guided fractionation and subsequent structure determination, the authors found that tannic acid and methyl and ethyl gallic acids possessed anti-HSV-1 activities with IC_50_ of 32, 20 and 26 μg/ml, respectively. Those compounds are known to bind to viral surface proteins to inhibit the virus infectivity, thereby exhibiting antiviral activities at the entry step. There have been no reports to date on the anti-HCV activities of *T. sureni* extracts, including TSL.

Chemical compounds of *M. latifolia, M. multiglandulosa* and *F. fistulosa* that possess antiviral activities have not been reported yet. An ethyl acetate extract of *M. vitiflora,* a plant genetically close to *M. latifolia,* was reported to possess antibacterial activities against methicillin-resistant *Staphylococcus aures* and *Micrococcus luteus*[18]*.* The authors identified some compounds contained in the extract, including coumarin and terpenoid compounds. Other Melicope species have been investigated for their chemical compounds. *M. triphylla* leaves were reported to contain 15 flavonoid compounds. Recently, Higa et al. [19] reported five flavonoids isolated from *M. triphylla* leaves; 5,8-dihydroxy-3,7dimethoxy-3,4-methylenedioxyflavone, 7-hydroxy-3,5-di-methoxy-3’,4’-methylenedioxyflavone, 7-(2,3-dihydroxy-3-methylbutoxy)-3,5-dimethoxy-3’,4’-methylene-dioxyflavone, 7-(2,3-dihydroxy-3-methylbutoxy)-3-3’,4’,5-tetramethoxyflavone, and 7-(2,3-dihydroxy-3-methylbutoxy)-3,3’4’,5,8-pentamethoxyflavone. There have been no reports to date on the antiviral activities of *M. latifolia* extracts, including MLL. On the other hand, many flavonoid compounds from plants have been reported to inhibit HCV replication [11,20].

*M. multiglandulosa* is a plant that belongs to the Euphorbiaceae family. There is no report so far regarding the possible antiviral activities of *M. multiglandulosa* extracts, including MMS. However, a butanol extract of another plant in the same family, *Excoecaria agallocha*, was reported to exert potential inhibitory effects on HCV NS3/4A protease [21]. Activity-guided fractionation and structure determination revealed that four polyphenol compounds of *E. agallocha*, such as excoecariphenol D, corilagin, geraniin and chebulagic acid, inhibited HCV NS3/4A protease activities and HCV RNA replication in cultured cells harbouring an HCV RNA replicon with IC_50_ of 12.6, 13.6, 33.2 and 22.3 μM, respectively.

*F. fistulosa* belongs to the genus *Ficus* in the family Moraceae. Many *Ficus* species have been used in folk medicine with various pharmacological actions against convulsion, respiratory disorder, tuberculosis and other infections. Plants from genus *Ficus* are rich sources of prenylated flavonoids, isoflavonoids, lignans, terpenoids, alkaloids and coumarins. Flavonoid compounds, such as β-amyrin, alpinum isoflavone, genistein, laburnetin, luteolin and catechin, isolated from *F. chlamydocarpa* and *F. cordata* were reported to have antibacterial and antifungal activities [22]. Also, antiviral activities against HSV-1, echovirus and adenovirus were detected in extracts of *F. carica*[23] and anti-HSV-1 activities in *F. binjamina*[24]. Bioassay-guided subfractionation of a flavonoid fraction of *F. benjamina* led to identification of three flavone glycosides; quersetin 3-O-rutinoside, kaempferol 3-O-rutinoside and kaempferol 3-O-robinobioside, which showed antiviral activities against HSV-1 with IC_50_ of 1.5 ± 0.56, 3.0 ± 0.97 and 0.9 ± 0.23 μM, respectively [25]. There have been no reports on anti-HCV activities in *F. fistulosa* extracts, including FFL.

The extracts of TSL, MLL, MMS and FFL may inhibit various steps of HCV life cycle. The viral life cycle can be divided into three major steps: (i) viral attachment and entry to the target cells, (ii) synthesis and processing of the viral proteins and replication of the viral genome, and (iii) assembly and release of the viral particles [1,2]. To explore the anti-HCV mechanisms of the plant extracts, time-of-addition analysis was performed in this study. The results obtained revealed that the extracts of TSL and MLL inhibited HCV infection at both the entry and post-entry steps whereas MMS and FFL extracts principally at the entry step (Table 2). In this connection, it should be noted that, despite the fact that the extracts of TSL and MLL inhibited HCV J6/JFH1 infection at the post-entry step, neither of them inhibited HCV RNA replication in an HCV-1b full-genomic RNA replicon system (data not shown). However, this does not necessarily rule out the possibility that these compounds may have proven efficacious if a genotype 2a replicon system had been used instead. This result rather suggests that the viral sensitivity to an antiviral plant extract(s) varies with different strains of HCV. Likewise, we observed that the sensitivity of the H77C/JFH1 strain to the FFL extract was much weaker compared to the other HCV strains (Table 3).

A flavonoid compound of green tea (*Camellia sinensis*), (−)-Epigallocatechin-3-gallate, was reported to inhibit HCV infection at the entry step with IC_90_ of 50 μM [26]. Another flavonoid from *Marrubium peregrinum L*, ladanein (BJ486K), inhibited the entry step, but not RNA replication or assembly, of HCV infection with IC_50_ of 2.5 μM [20]. On the other hand, silymarin, an extract of *Silybum marianum*, was reported to inhibit HCV entry, replication and cell-to-cell transmission with IC_50_ of 40 to 100 μM [27]. Silibinin, the major component of silymarin consisting of two flavonolignans, silibinin A and silibinin B, has currently been used to prevent reinfection of the graft after liver transplantation [28]. An increasing body of information on natural compounds possessing anti-HCV activities is summarized elsewhere [6]. As for the TSL, MLL, MMS and FFL, further analyses will be needed to determine the possible anti-HCV compounds present in their extracts. In this connection, we observed in a preliminary experiment that TSL, MLL, MMS and FFL showed anti-measles virus activities (data not shown). This result suggests that the compounds present in the extracts inhibit viral and/or cellular machineries commonly used for replication of different viruses. This should also be clarified by further mechanistic studies.

## Conclusions

Ethanol extracts of *Toona sureni* leaves (TSL), *Melicope latifolia* leaves (MLL), *Melanolepis multiglandulosa* stem (MMS), and *Ficus fistulosa* leaves (FFL) inhibited the hepatitis C virus (HCV) J6/JFH1-P1 and P-47 strains with IC_50_ ranging between 2.0 and 17.1 μg/ml. All of the HCV genotypes 1a to 7a, 1b and 2b were inhibited by the plant extracts to the same extent, with the exception that the H77C/JFH1 strain of HCV genotype 1a showed significant resistance to FFL, and the J8/JFH1 (2b) and QC69/JFH1 strains (7a) to MMS to lesser extents. As for the mode of action, TSL and MLL inhibited HCV infection both at the entry and post-entry steps while MMS and FFL principally at the entry step.

## Materials and methods

### Cells and viruses

Huh7.5 cells and the plasmid pFL-J6/JFH1 to produce the J6/JFH1 strain of HCV genotype 2a [15] were kindly provided by Dr. C. M. Rice, The Rockefeller University, New York, NY. The plasmid for the original JFH1 strain [16] was kindly provided by Dr. T. Wakita, National Institute of Infectious Diseases, Tokyo, Japan and those for other HCV genotypes, pH77C/JFH1 (1a), pJ4/JFH1 (1b), pJ8/JFH1 (2b), pS52/JFH1 (3a), pED43/JFH1 (4a), pSA13/JFH1 (5a), pHK6a/JFH1 (6a) and pQC69/JFH1 (7a) [3], were kindly provided by Dr. J. Bukh, Copenhagen University Hospital, Hvidovre, Denmark. Huh7.5 cells were cultivated in Dulbecco’s modified Eagle’s medium (Wako, Osaka, Japan) supplemented with fetal bovine serum (Biowest, Nuaille, France), non-essential amino acids (Invitrogen, Carlsbad, CA), penicillin (100 IU/ml) and streptomycin (100 μg/ml) (Invitrogen). Cells were grown at 37°C in a 5% CO_2_ incubator.

### Collection and extraction of medicinal plants

Seventeen species of medicinal plants were collected at Cangar forest, the East Java region of Indonesia. The plants collected were verified by botanical researchers at Purwadadi Botanical Garden, Purwadadi, Indonesia. Parts of the plants were dried at room temperature and pulverized on the basis of their characteristics. They were macerated in 80% ethanol for overnight to extract constituents. After 24 hours, the extracts were filtered and the residue was soaked again in fresh solvents. The filtration process was repeated over 3 days. The filtrates were evaporated by using an evaporator at temperature not exceeding 40°C. The extracts for bioassay were dried in vacuo before being used.

### Sample stock preparations

The dried ethanol extracts were weighed 10.0 mg and suspended in 100 μl of dimethyl sulfoxide (DMSO) to obtain stock solutions at a concentration of 100 mg/ml. The stock solutions were stored at −20°C until used.

### Analysis of anti-HCV activities of plant extracts

Huh7.5 cells were seeded in 24-well plates (1.9 × 10^5^ cells/well). A fixed amount of HCV was mixed with serial dilutions of medicinal plant extracts (500, 100, 50, 10 and 1 μg/ml) and inoculated to the cells. After 2 hours, the cells were washed with medium to remove the residual virus and further incubated in the medium containing the same concentrations of the plant extracts as those during virus inoculation. In some experiments, treatment with medicinal plant extracts was done only during virus inoculation or only after virus inoculation for the remaining period of the culture until virus harvest in order to assess the mode of action of the plant extracts. Culture supernatants were obtained at 1 and 2 days post-infection (dpi) and titrated for virus infectivity expressed as focus-forming units/ml, as described previously [14]. Virus and cells treated with medium containing 0.1% DMSO served as a control. Percent inhibition of the virus infectivity by the samples was calculated by comparing to the control by using SPSS probit analysis, and IC_50_ values were determined. Percent inhibition of the compounds at the concentration of 30 μg/ml was determined also for the other genotypes of HCV.

### Immunoblotting

Cells were lysed with SDS sample buffer, and equal amounts of protein were subjected to SDS-polyacrylamide gel electrophoresis and transferred onto a polyvinylidene difluoride membrane (Millipore, Bedford, MA), which was then incubated with the respective primary antibody. The primary antibodies used were mouse monoclonal antibodies against HCV NS3 and glyceraldehyde-3-phosphate dehydrogenase (GAPDH) (Millipore). Horseradish peroxidase-conjugated goat anti-mouse immunoglobulin (Invitrogen) was used to visualize the respective proteins by means of an enhanced chemiluminescence detection system (ECL; GE Healthcare, Buckinghamshire, UK).

### Real-time quantitative RT-PCR

Total RNA was extracted from the cells using a ReliaPrep RNA cell miniprep system (Promega, Madison, WI) according to the manufacturer’s instructions. One μg of total RNA was reverse transcribed using a GoScript Reverse Transcription system (Promega) with random primers and was subjected to quantitative real-time PCR analysis using SYBR Premix Ex Taq (TaKaRa, Kyoto, Japan) in a MicroAmp 96-well reaction plate and an ABI PRISM 7500 system (Applied Biosystems, Foster City, CA). The primers used to amplify an NS5A region of the HCV genome were 5’-AGACGTATTGAGGTCCATGC-3’ (sense) and 5’-CCGCAGCGACGGTGCTGATAG-3’ (antisense). As an internal control, human GAPDH gene expression levels were measured using primers 5’-GCCATCAATGACCCCTTCATT-3’ (sense) and 5’-TCTCGCTCCTGGAAGATGG-3'.

### WST-1 assay for cytotoxicity

WST-1 assay was performed as described previously with a slight modification [29]. In brief, Huh7.5 cells in 96-well plates were treated with serial dilutions of the medicinal plant extracts or 0.1% DMSO as a control for 48 hours. After the treatment, 10 μl of WST-1 reagent (Roche, Mannheim, Germany) was added to each well and cells were cultured for 4 hours. The WST-1 reagent is absorbed by the cells and converted to formazan by mitochondrial dehydrogenases. The amount of formazan, which correlates with the number of living cells, was determined by measuring the absorbance of each well using a microplate reader at 450 nm and 630 nm. Percent cell viability compared to the control was calculated for each dilution of the plant extracts and CC_50_ values were determined by SPSS probit analysis.

## Abbreviations

CC50: 50% cytotoxic concentration; DMSO: Dimethyl sulfoxide; FFL: *Ficus fistulosa* leaves; HCV: Hepatitis C virus; IC50: 50% inhibitory concentration; MMS: *Melanolepis multiglandulosa* stem; MLL: *Melicope latifolia* leaves; TSL: *Toona sureni* leaves.

## Competing interests

The authors declare that they have no competing interests.

## Authors’ contributions

TSW, AAP, EA, MA, IS, LD and CA contributed to anti-HCV bioassay work. TSW, LT, AW, AR and AF contributed to exploration and phytochemistry work. HF and NK participated in phytochemistry work. MIL, S, N, CA and HH, as principle investigators, planned and coordinated the study. TSW, LD and HH wrote the manuscript. All the authors read and approved the final manuscript.
